# LSDP5 Enhances Triglyceride Storage in Hepatocytes by Influencing Lipolysis and Fatty Acid β-Oxidation of Lipid Droplets

**DOI:** 10.1371/journal.pone.0036712

**Published:** 2012-06-01

**Authors:** Hang Li, Yue Song, Li-Jun Zhang, Yu Gu, Fan-Fan Li, Shu-Yi Pan, Li-Na Jiang, Fang Liu, Jing Ye, Qing Li

**Affiliations:** 1 State Key Laboratory of Cancer Biology and Department of Pathology, Xijing Hospital, Fourth Military Medical University, Xi’an, China; 2 Department of Clinical Laboratory, Tangdu Hospital, Fourth Military Medical University, Xi’an, China; 3 Hyperbaric Oxygen Center of Chinese People’s Liberation Army, Navy General Hospital, Beijing, China; Chinese University of Hong Kong, Hong Kong

## Abstract

Lipid storage droplet protein 5 (LSDP5) is a lipid droplet-associated protein of the PAT (perilipin, adipophilin, and TIP47) family that is expressed in the liver in a peroxisome proliferator-activated receptor alpha (PPARα)-dependent manner; however, its exact function has not been elucidated. We noticed that LSDP5 was localized to the surface of lipid droplets in hepatocytes. Overexpression of LSDP5 enhanced lipid accumulation in the hepatic cell line AML12 and in primary hepatocytes. Knock-down of LSDP5 significantly decreased the triglyceride content of lipid droplets, stimulated lipolysis, and modestly increased the mitochondrial content and level of fatty-acid β-oxidation in the mitochondria. The expression of PPARα was increased in LSDP5-deficient cells and required for the increase in the level of fatty acid β-oxidation in LSDP5-deficient cells. Using serial deletions of LSDP5, we determined that the lipid droplet-targeting domain and the domain directing lipid droplet clustering overlapped and were localized to the 188 amino acid residues at the N-terminus of LSDP5. Our findings suggest that LSDP5, a novel lipid droplet protein, may contribute to triglyceride accumulation by negatively regulating lipolysis and fatty acid oxidation in hepatocytes.

## Introduction

Obesity occurs because of an imbalance between energy intake and expenditure. Most excess energy is stored as triglycerides (TGs) in lipid droplets in adipose tissue. Overaccumulation of lipid droplets in non-adipose tissues, such as in the liver, pancreatic islets, and coronary artery, is often associated with fatty liver, type 2 diabetes, and coronary atherosclerotic heart disease [1], [2], [3]. However, the mechanisms of lipid droplet formation in these tissues remain poorly understood.

Lipid droplets are structurally similar to circulating lipoproteins as both have a core of esterified lipids (primarily TGs, cholesterol esters, retinol esters, or other lipids depending on the cell type) that is encased by a phospholipid monolayer and a coat of proteins [4]. The protein components associated with lipid droplet surfaces are called lipid droplet-associated proteins. Lipid droplet-associated proteins are involved in the formation, maturation, secretion, and trafficking of lipid droplets and participate in regulating of lipid metabolism in cells, including both lipolysis and lipogenesis [5], [6], [7], [8]. The best characterized lipid droplet-associated protein is perilipin, which shares sequence similarity with two other lipid droplet-associated proteins, adipophilin/adipocyte differentiation-related protein (ADRP) and tail-interacting protein 47 (TIP47). Together, these proteins form the PAT (perilipin-adipophilin-TIP47) family of proteins, and S3-12 has recently been classified in this family [6]. Perilipin is a phosphoprotein involved in hormone-stimulated lipolysis, and its expression is restricted to adipocytes [9]. Adipophilin is ubiquitously expressed, and functions in limiting the interaction of lipases with the neutral lipids within droplets, which promotes neutral lipid accumulation [6]. TIP47 and S3-12 coat smaller lipid droplets, where it is possible that they participate in the early events of lipid droplet formation [6], [10]. Lipid storage droplet protein 5 (LSDP5)/perilipin-5 is a newly identified member of the PAT family. The initial identifications and characterizations of LSDP5 as a lipid droplet-binding protein were reported by three independent groups, who named the protein myocardial lipid droplet protein (MLDP), oxidative tissue-enriched PAT protein (OXPAT), and LSDP5 [11], [12], [13]. These studies reported that LSDP5 is ubiquitously expressed in tissues that exhibit high levels of fatty acid oxidation, including the heart, skeletal muscle, and liver. LSDP5 RNA and/or protein are induced in the heart, liver, and skeletal muscle by fasting and in gastrocnemius muscle by a high-fat diet [11], [12], [13], [14]. Similar to other members of the PAT family, the expression of LSDP5 is regulated by peroxisome proliferator-activated receptor α (PPARα), a ligand-activated transcription factor belonging to the nuclear receptor superfamily [6], [11], [12], [13], [15]. Stable heterologous expression of LSDP5 is associated with increased TG accumulation in oleate-treated COS-7 and OP9 cells [11], [13]. To date, the functional evaluation of LSDP5 has been limited to gain-of-function studies in cultured cells. No loss-of-function studies by gene knockout or by RNAi have been reported. The mechanisms by which LSDP5 promotes the lipid accumulation are also mostly unknown.

LSDP5 is expressed in the liver [11], [12], [13], which plays a central role in energy homeostasis because it is the primary organ of de novo lipid synthesis, lipid uptake and secretion, fatty acid oxidation, and production of ketone bodies. In the present study, we investigated the function of LSDP5 in murine hepatocytes (in the AML12 cell line and primary mouse liver cells). Our results provide evidence that LSDP5 is targeted to lipid droplets and plays an important role in lipid accumulation. This study also reveals the mechanisms by which LSDP5 promotes TG deposition in lipid droplets.

## Results

### LSDP5 Localizes to Lipid Droplets in Hepatocytes

**Figure 1:**
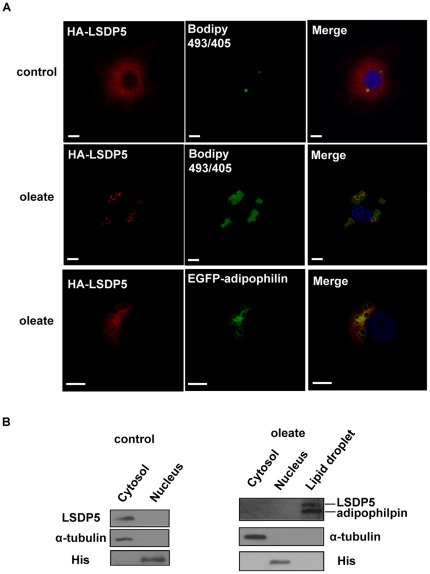
LSDP5 was recruited to lipid droplets. (A) AML12 cells were transiently transfected with HA-tagged LSDP5 and incubated with BSA (control) or oleate (100 µM) overnight. The cells were stained with an anti-HA antibody with BODIPY 493/503 for visualizing lipids (green) and Hoechst 33258 for visualizing nuclei (blue). Left panels show the immunofluorescent signal (red), middle panels showed BODIPY staining (green), and right panels show the merged images. HA-LSDP5 exhibited a steady-state faint cytoplasmic staining and decorated lipid droplets after incubating the cells in oleate-rich medium. AML12 cells were co-transfected with HA-LSDP5 and EGFP-adipophilin. The cells were incubated with a mouse anti-HA antibody (primary antibody) and a Cy3-conjugated anti-mouse antibody (secondary antibody). The samples were detected by fluorescence microscopy (Olympus, Temecula, CA). The results show the co-localization of LSDP5 with adipophilin, a lipid droplet-targeted protein (last row). Scale bar = 5 µm. (B) LSDP5 was enriched in lipid droplet fractions. α-tubulin, a cytosol marker; His, a nucleus marker; and adipophilin, a lipid droplet marker. 5 mg of each fraction was loaded for immunoblot analysis.

Little is currently known about the subcellular localization of LSDP5 in liver cells. A vector containing hemagglutinin (HA)-tagged LSDP5 was transfected into AML12 cells and oleate was used to promote the enlargement of lipid droplets. As shown in Figure 1A, HA-LSDP5 staining was visible throughout the AML12 cells in the absence of oleate. After oleate was added into the culture medium, HA-LSDP5 staining showed a distinct ring pattern surrounding the cores of neutral lipids. LSDP5 also co-localized with enhanced green fluorescent protein (EGFP)-adipophilin, which is a well-recognized marker of lipid droplets. To provide further evidence of the intracellular location of LSDP5, we investigated the subcellular localization of LSDP5 by biochemical subcellular fractionation and Western blot analysis. The majority of the LSDP5 protein was detected in the cytosol under normal conditions, whereas it was mainly detected in the lipid droplet fraction and cofractionated with adipophilin upon treatment with oleate (Figure 1B).

### The Dynamics of LSDP5 Expression During TG Accumulation

**Figure 2:**
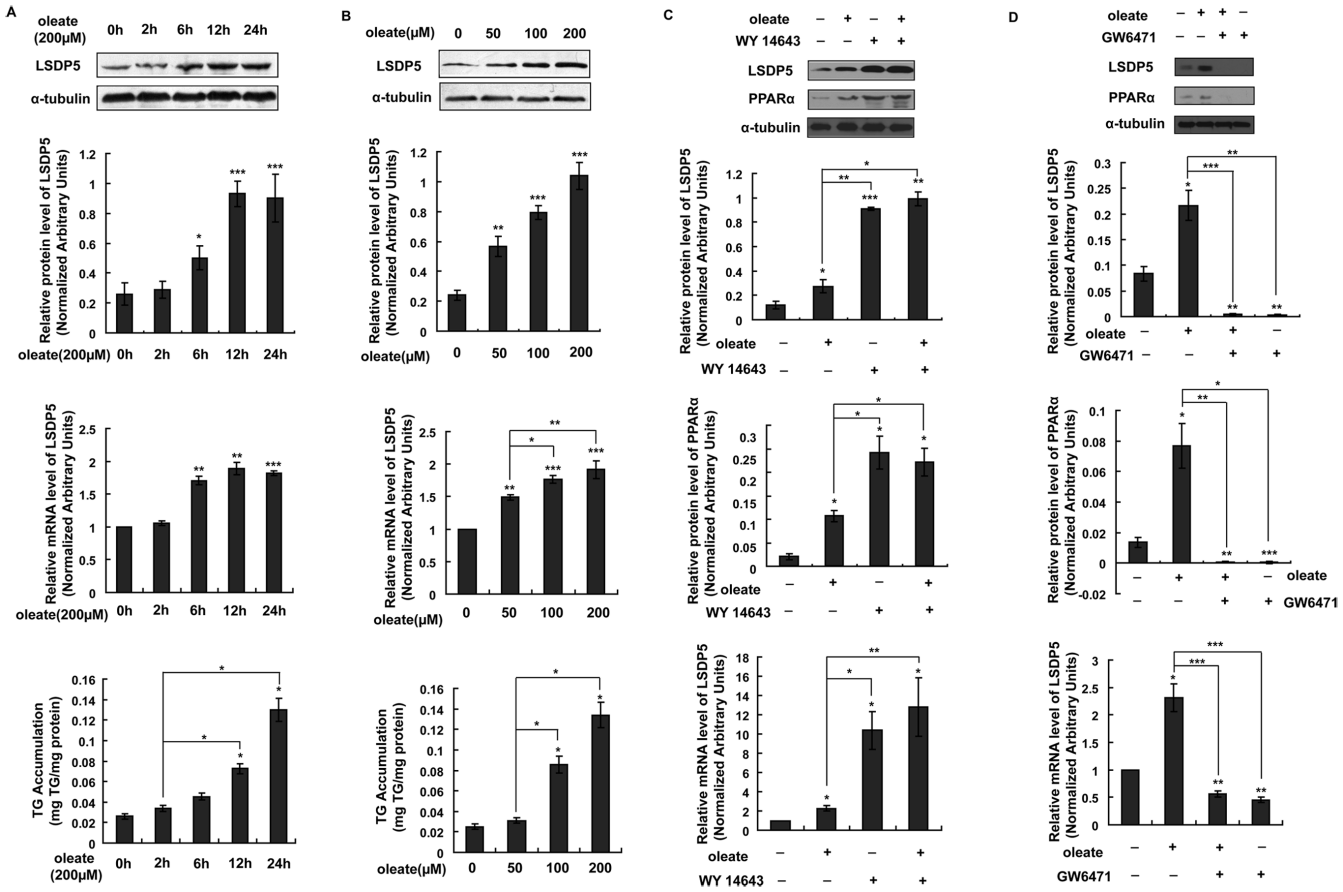
The effect of oleate exposure on the expression of LSDP5. (A) AML12 cells were incubated with 200 µM oleate for the indicated time. Representative Western blots are shown (top panel), and LSDP5 transcript levels were measured using real-time PCR (middle panel). Expression levels of LSDP5 are expressed as a ratio to α-tubulin (representative of three experiments). ^*^ *P*<0.05, ^**^ *P*<0.01, ^***^ *P*<0.001. The relative mRNA level before oleate exposure (0 h) was designated as 1.0. n = 6, ^**^ *P*<0.01, ^***^ *P*<0.001. The amount of TGs in AML12 cells treated with oleate for different times was assessed with a TG test kit, and is expressed as mg TG/mg protein (bottom panel). n = 5, ^*^ *P*<0.05, ^**^ *P*<0.01, ^***^ *P*<0.001. Data are presented as the mean±SEM. (B) AML12 cells were incubated with different concentrations of oleate (0, 50, 100, and 200 µM) for 24 h. Protein extracts were analyzed by Western blotting (top panel), and total RNA was subjected to real-time PCR (middle panel). n = 6,^*^ *P*<0.05, ^**^ *P*<0.01, ^***^ *P*<0.001. The amount of TGs in AML12 cells treated with different concentration of oleate was assessed with a TG test kit (bottom panel). n = 5, ^*^ *P*<0.05. Data are presented as the mean±SEM. (C,D) Effect of WY14643 or GW6471 on oleate-induced LSDP5 expression in AML12 cells. AML12 cells were exposed to oleate (200 µM) in the absence or presence of WY 14643 (30 µM) or GW6471 (10 µM) for 24 h. The expression of LSDP5 (top panel) and PPARα (middle panel) was monitored by Western blotting. LSDP5 transcript levels were measured using real-time PCR (bottom panel). The relative mRNA level of AML12 cells in equivalent amounts of BSA was designated as 1.0. Data are presented as the mean±SEM (n = 4–6), ^*^ *P*<0.05, ^**^ *P*<0.01, ^***^ *P*<0.001 (Dunnett’s post hoc test following a one-way ANOVA).

The expression and subcellular localization of PAT family members vary over time during the process of lipid droplet biogenesis and enlargement [10], [16], [17]. However, little is known about the specific changes of LSDP5 during this process. The expression levels of LSDP5 were monitored during TG accumulation (Figure 2A&2B). AML12 cells were exposed to oleate, which provided the substrate for TG synthesis. As shown in Figure 2A, the transcriptional level of LSDP5 did not significantly change 2 h after oleate treatment (*P* = 0.191). However, an increase in LSDP5 mRNA was detectable 6 h after incubation with oleate, and the level of LSDP5 mRNA was markedly increased after 12 h. The LSDP5 mRNA level remained high after prolonged incubation with oleate (24 h). The amount of LSDP5 protein increased in parallel with the observed increases in its mRNA level, and the expression of LSDP5 increased in a dose-dependent manner upon treatment with oleate (Figure 2B). However, the TG content of the AML12 cells did not exhibit a significant positive correlation with the transcriptional level of LSDP5 (r = 0.826, *P* = 0.085 at different times [0, 2, 6, 12, or 24 h] after oleate exposure and r = 0.905, *P* = 0.095 at different concentrations [0, 50, 100, or 200 µM]).

Free fatty acids can potentially activate PPARα expression and PPARα regulates LSDP5 transcription [12], [13]. It remains unknown whether free fatty acids induce LSDP5 by modulating PPARα activity or through a PPARα-independent mechanism. We detected the expression of LSDP5 in the presence of the PPARα agonist (WY14643) as well as in the presence of the PPARα inhibitor (GW6471) in the absence or presence of oleate treatment (200 µM, 24 h). As shown in Figure 2C&2D, exposure of the cells to WY14643 led to increased expression of LSDP5 regardless whether oleate was administered. By inhibiting PPARα, GW6471 completely abrogated increased LSDP5 expression in response to oleate stimulation. These results indicate that the oleate-induced LSDP5 increase in expression is regulated in a PPARα-dependent manner.

### LSDP5 Stimulates the Storage of TG within the Lipid Droplets of Hepatocytes

**Figure 3:**
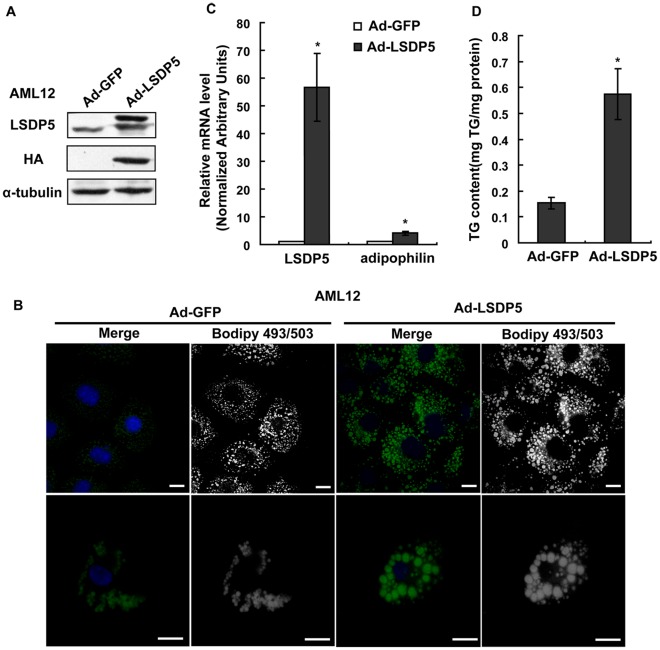
The overexpression of LSDP5 increased cellular TG storage in AML12 cells. (A) Adenovirus-mediated HA-LSDP5 overexpression in AML12 cells was confirmed by Western blotting. Expression levels of LSDP5 are expressed as a ratio to α-tubulin (representative of three experiments). (B) AML12 cells were infected with adenovirus encoding LSDP5 for 6 h and then incubated with 200 µM oleate for 24 h. Neutral lipids were stained with BODIPY 493/503, and nuclei were labeled with Hoechst 33258. The immunofluorescent signals of both BODIPY 493/503 (green) and Hoechst 33258 (blue) are shown in the merged panels. The BODIPY immunofluorescent signals are shown in the BODIPY493/503 panels and were edited to grayscale to provide clearer lipid signals and reduce interference from the immunofluorescent signal of the nucleus. Scale bar = 15 µm. (C) The relative mRNA levels of LSDP5 and adipophilin were assessed using real-time PCR. Data are presented as the mean±SEM (n = 4), ^*^ *P*<0.05. (D) A higher concentration of TGs was observed in cells overexpressing LSDP5 compared with controls. Data are presented as the mean±SEM (n = 5),^ *^ *P*<0.05. Data in this figure were analyzed with paired Student’s *t* test.

To facilitate the study of LSDP5 in lipid metabolism, LSDP5 was overexpressed using an efficient adenovirus expression system both in the AML12 mouse hepatic cell line (Figure 3A) and in primary mouse hepatocytes (Figure S1A). As shown in Figure 3B, lipid droplets were stained using BODIPY (neutral lipid dye). BODIPY fluorescence increased in cells overexpressing LSDP5. The mRNA level of adipophilin, a protein that coats lipid droplets, was increased in cells overexpressing LSDP5 (Figure 3C). The amount of TGs in cells overexpressing LSDP5 also significantly increased, compared to control cells, which was determined using a TG test kit (*P* = 0.014) (Figure 3D). The phenotypes of increased lipid droplet storage and increased TG contents were also observed in primary hepatocytes (Figure S1). These data demonstrate that the overexpression of LSDP5 is associated with an increased cellular TG content.

### LSDP5 Deficiency Inhibits Lipid Droplet Storage in Hepatocytes

**Figure 4:**
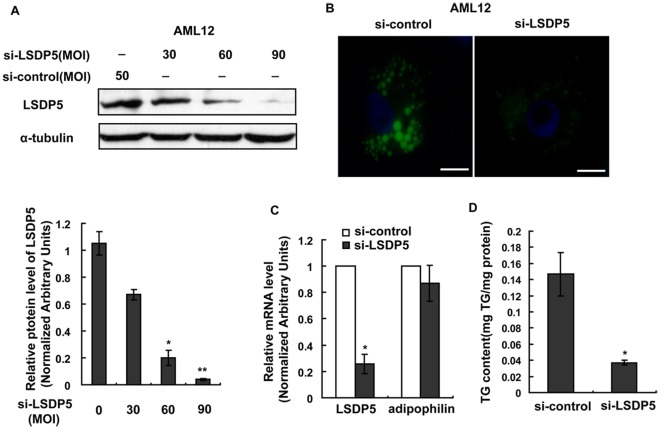
LSDP5 deficiency inhibited TG accumulation in AML12 cells. AML12 cells were infected with an adenovirus carrying LSDP5 siRNA for 24 h and then incubated with 200 µM oleate for another 24 h. (A) Western blotting revealed that the adenovirus (si-LSDP5) at a multiplicity of infection (MOI) of 90 successfully silenced LSDP5 in AML12 cells (>95% knock-down). Expression levels of LSDP5 are expressed as a ratio to α-tubulin (representative of three experiments). Data are presented as the mean±SEM. ^*^ *P*<0.05, ^**^ *P*<0.01 (Dunnett’s post hoc test following a one-way ANOVA). (B) BODIPY staining of AML12 cells expressing control siRNA (left) or LSDP5 siRNA (right). Scale bar = 15 µm. (C) The mRNA levels of LSDP5 and adipophilin were assessed with real-time PCR. The relative mRNA level in AML12 cells infected with an adenovirus containing control siRNA was designated as 1.0. Data are presented as the mean±SEM (n = 4), ^*^ *P*<0.05. (D) A lower concentration of TGs was detected in si-LSDP5 cells, compared with control cells. Data are presented as the mean±SEM (n = 5), ^*^ *P*<0.05 (paired Student’s *t* test).

The expression of LSDP5 was knocked down in AML12 cells (Figure 4A) and in primary mouse hepatocytes (Figure S2A) using an adenovirus-mediated gene silencing approach to investigate the effect of LSDP5 deficiency on cell morphology. BODIPY fluorescence in LSDP5-deficient cells was less than that in control cells under lipid loading (Figure 4B). The depletion of LSDP5 significantly reduced the TG content of AML12 cells (*P* = 0.016), which was analyzed using a TG test kit (Figure 4D). Decreases in BODIPY fluorescence and TG levels were also measured in primary mouse hepatocytes upon depletion of LSDP5 (Figure S2). To further confirm these results, a cell line was established where LSDP5 was stably knocked down (AML12-si-LSDP5) (Figure S3A&S3B). As shown in Figure S3C, the BODIPY-positive signal was reduced in AML12-si-LSDP5 cells, and the cellular TG content was also significantly lowered (*P* = 0.010) (Figure S3D).

Collectively, the data from the overexpression and silencing experiments indicate that LSDP5 plays an important role in TG accumulation in liver cells.

### Effects of LSDP5 Deficiency on TG Metabolism

**Figure 5:**
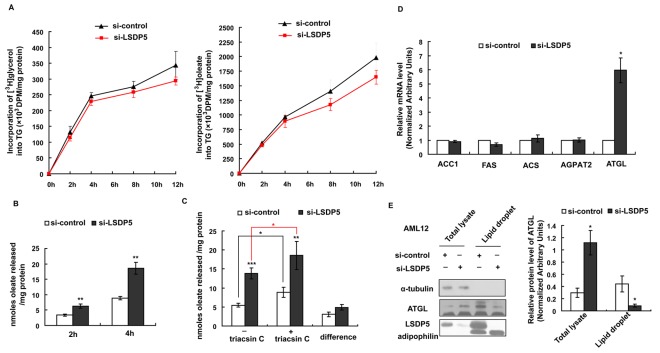
Triglyceride synthesis and lipolysis in AML12 cells lacking LSDP5. (A) Incorporation of [^3^H]-glycerol or [^3^H]-oleate into TG in control and si-LSDP5 cells. Data were normalized to the number of cells and are expressed as the mean±SEM (n = 4). (B) Time courses of [^3^H]-oleate release from si-control and si-LSDP5 cells. Trypan blue staining was used to count the living cells. Data were normalized based on cell viability and total protein content. Data are presented as the mean±SEM (n = 4), ^**^ *P*<0.01. (C) The effect of LSDP5 silencing on re-esterificaiton and hydrolysis. After lipid loading, the [^3^H]-oleate release after 4 h with or without triacsin C was assessed. The efflux of [^3^H]-oleate without triacsin C reflected the total lipolysis level, the efflux of [^3^H]-oleate with triacsin C reflected the TG hydrolysis level, and the difference between TG hydrolysis and total lipolysis reflected the level of re-esterification. Data were normalized based on cell viability and total protein level. Data are presented as the mean±SEM (n = 4–5), ^*^ *P*<0.05. (D) The mRNA levels of ACC1, FAS, ACS, AGPAT and ATGL were assessed using real-time PCR. The relative mRNA level in the control group was designated as 1.0. Data are presented as the mean±SEM (n = 6), ^*^ *P*<0.05. (E) Lipid droplet fractions were isolated by subcellular fractionation and analyzed by immunoblotting for LSDP5, adipophilin, and ATGL. 20 mg of total hepatocyte lysate and 5 mg of lipid fraction were loaded for immunoblotting analysis. Expression levels of ATGL are expressed as a ratio to α-tubulin in the total lysate and as a ratio to adipophilpin on lipid droplets (representative of four experiments). Data are presented as the mean±SEM, ^*^ *P*<0.05. Data in this figure were analyzed with paired Student’s *t* tests.

An appropriate balance between TG synthesis and lipolysis is crucial for maintaining lipid homeostasis, and an imbalance between TG synthesis and lipolysis may result in TG accumulation [3]. AML12 cells were infected with an adenovirus carrying LSDP5 siRNA (MOI = 90) for 24 h and then incubated with radiolabeled tracers. As shown in Figure 5A, there was no significant difference in the incorporation of radiolabeled glycerol or oleate precursor into TGs between the si-LSDP5 and the control cells, which suggests that the synthesis of TGs was not affected by LSDP5 silencing.

To determine the rate of lipolysis, AML12 cells lacking LSDP5 were loaded with [^3^H]-oleate for 24 h. After the loading period, the [^3^H]-labeled TGs accounted for 77±6.7% and 80±7.9% of the total TG content in the si-LSDP5 and si-control cells, respectively. There was no significant difference between the si-LSDP5 and si-control cells (*P* = 0.557), which demonstrated that [^3^H]-oleate was successfully incorporated into TGs after the loading period. The efflux of [^3^H]-oleate to the medium was monitored for 4 h and reflects the rate of lipolysis in cells. Triacsin C was administrated to block the re-esterification. As shown in Figure 5B, oleate release was increased up to 2-fold in AML12 cells lacking LSDP5 after 2 h (*P* = 0.002) and 4 h (*P* = 0.008); indicating that LSDP5 silencing up-regulated the lipolysis level.

Lipolysis is the total of TG hydrolysis and re-esterification. We used triacsin C to block re-esterification in order to determine the component of lipolysis that is affected by si-LSDP5. As shown in Figure 5C, knock-down of LSDP5 equally increased lipolysis in the presence or absence of triacsin C, which indicates that LSDP5 modulates TG hydrolysis and does not affect re-esterification.

Acetyl-CoA carboxylase 1 (ACC1), fatty acid synthase (FAS), 1-O-acylceramide synthase (ACS), 1-acyl-sn-glycerol-3-phosphate acyltransferase β (AGPAT2) and adipose triglyceride lipase (ATGL) have been reported to be crucial to TG metabolism in vitro and liver steatosis in vivo [18]. Therefore, these genes were tested in this study. When LSDP5 was knocked down, there was no significant change in the mRNA levels of ACC1 and FAS, the key enzymes in fatty acid synthesis, or ACS and AGPAT2, the enzymes that regulated the TG synthesis. However, knock-down of LSDP5 increased the mRNA level of ATGL, an important lipolysis enzyme (Figure 5D). LSDP5 silencing increased the ATGL protein level in the total lysate but decreased the concentration of ATGL on lipid droplets (Figure 5E).

These data indicate that the loss of LSDP5 increases lipolysis in liver cells, especially TG hydrolysis.

### Effects of LSDP5 Deficiency on Fatty Acid β-Oxidation in Mitochondria

**Figure 6:**
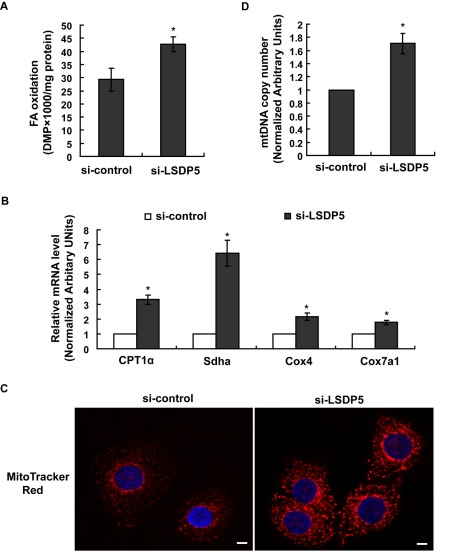
Depletion of LSDP5 increased fatty acid β-oxidation and the number of mitochondria. AML12 cells were infected with adenovirus carrying LSDP5 siRNA (MOI = 90) or an adenovirus carrying control siRNA for 24 h. (A) β-oxidation of [^3^H]-oleate in si-control or si-LSDP5 cells. Data are presented as the mean±SEM (n = 4), ^*^P<0.05. (B) The mRNA levels of CPT1α, Sdha, Cox4 and Cox7a1 were assessed using real-time PCR. The relative mRNA level in the control group was designated as 1.0. Data are presented as the mean±SEM (n = 5), ^*^P<0.05. (C) Mitochondria in hepatocytes containing control-siRNA or LSDP5-siRNA were stained with MitoTracker Red. Nuclei were labeled with Hoechst 33258. Scale bar = 5 µm. ^*^P<0.05. (D) Changes in the copy number of mtDNA were assessed with real-time PCR. The relative amount of mtDNA was calculated as the normalized ratio of NADH dehydrogenase subunit I/lipoprotein lipase, and the relative mtDNA copy number in the control group was designated as 1.0. Data are presented as the mean±SEM (n = 4), ^*^ *P*<0.05. Data in this figure were analyzed with paired Student’s *t* tests.

Mitochondrial fatty acid β-oxidation was studied in LSDP5-depleted AML12 cells. The oxidation of oleate was assayed by analyzing the oxidation products in the media after the cells were labeled with radioactive oleate. In cells with LSDP5 depletion, we observed an increase in oxidation of labeled oleate (Figure 6A). Real-time PCR analyses revealed that knock-down of LSDP5 increased the mRNA level of carnitine palmitoyltransferase1α (CPT1α) (the rate-limiting enzyme in fatty acid β-oxidation) and a-subunit of succinate dehydrogenase (Sdha) (an enzyme in long-chain fatty acid oxidation) (Figure 6B). By labeling the mitochondria with MitoTracker Red, we observed that the mitochondrial signal was greater in LSDP5-depleted cells compared with the control cells (Figure 6C). The amount of mitochondrial DNA (mtDNA) also increased in LSDP5-depleted AML12 cells compared with that in the control group (Figure 6D). In LSDP5 knock-down cells, we observed significant up-regulation of the mRNA levels of cytochrome c oxidase subunit IV (Cox4) and cytochrome c oxidase subunit VIIa polypeptide 1 (Cox7a1); both of these genes encode components of the mitochondrial respiratory chain (Figure 6B). This finding is consistent with the observation that the number of mitochondria increase in LSDP5-deficient cells.

### PPARα is Required for the Increase in Datty Acid Oxidation in LSDP5-Deficient Cells

**Figure 7:**
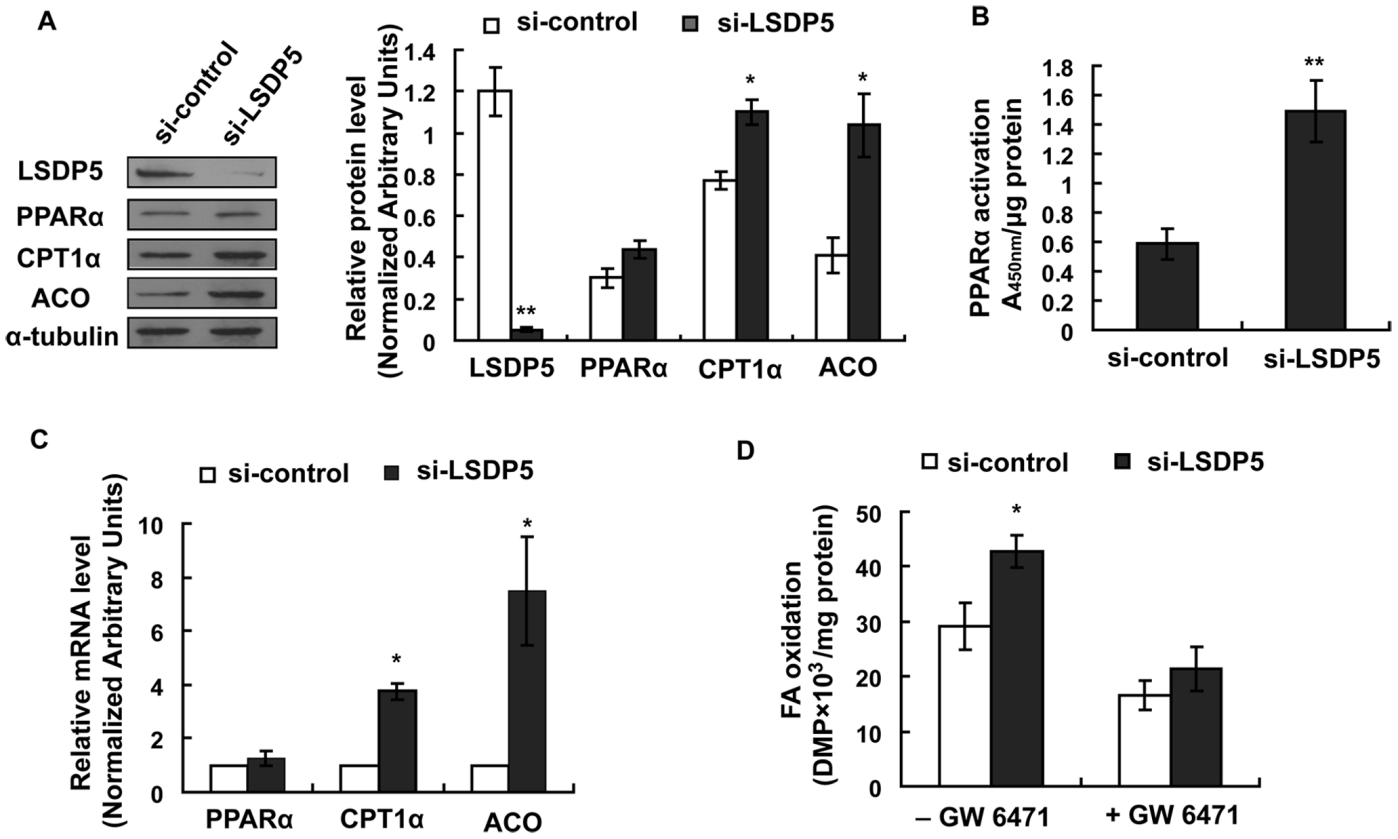
Effect of LSDP5 silencing on PPARα expression and activity. (A) A representative immunoblot for PPARα, LSDP5, CPT1α and ACO as detected by chemiluminescence. Expression levels of each protein are expressed as a ratio to α-tubulin (representative of four experiments). Data are presented as the mean±SEM, ^*^ *P*<0.05,^ **^ *P*<0.01. (B) PPARα activity was determined by a binding assay using nuclear protein from si-control and si-LSDP5 cells and an oligonucleotide corresponding to the PPARα consensus sequence. The activity of PPARα significantly increased when LSDP5 was knocked down. The results are presented as the absorbance at 450 nm (*A450*) wave length/µg protein. Data are presented as the mean±SEM (n = 5), ^*^ *P*<0.05. (C) Quantitative analysis of the mRNA level of using LSDP5, CPT1α and ACO as determined by real-time PCR and expressed relative to the corresponding siRNA control group. Data are presented as the mean±SEM of three independent experiments. (D) Effect of GW6541 on increased β-oxidation in LSDP5-depleted hepatocytes. Data are presented as the mean±SEM (n = 5), ^*^ *P*<0.05. Data in this figure were analyzed with paired Student’s *t* tests.

PPARα plays a critical role in stimulating fatty acid oxidation in the liver [15]. LSDP5 has been shown to be a PPARα target gene, and its expression depends both on the physiological conditions and action of PPARα [13]. We hypothesized that down-regulation of LSDP5 might stimulate PPARα and that the increased level of fatty acid β-oxidation observed in LSDP5-deficient hepatocytes might be due to an up-regulation of PPARα. Although the mRNA and protein levels of PPARα were not significantly different between the si-control and si-LSDP5 groups (Figure 7A&7C), the PPARα activity was nearly 3-fold greater in the LSDP5-deficient cells than in the control cells (Figure 7B). The expression levels of two classical PPARα target genes, CPTIα and acyl CoA oxidase (ACO), were significantly increased when LSDP5 was knocked down (Figure 7A&C). The level of oleate oxidation was increased in LSDP5-deficient cells. However, when PPARα was inhibited by GW6471, the level of fatty acid oxidation was not significantly different between the si-LSDP5 and si-control cells (Figure 7D), indicating that PPARα was required for the increase in the level of fatty acid oxidation in LSDP5-deficient cells. These data show that increased fatty acid β-oxidation in LSDP5-deficient cells is mediated by PPARα activation.

### The N-Terminus of LSDP5 is Essential for TG Accumulation and Lipid Droplet Targeting

**Figure 8:**
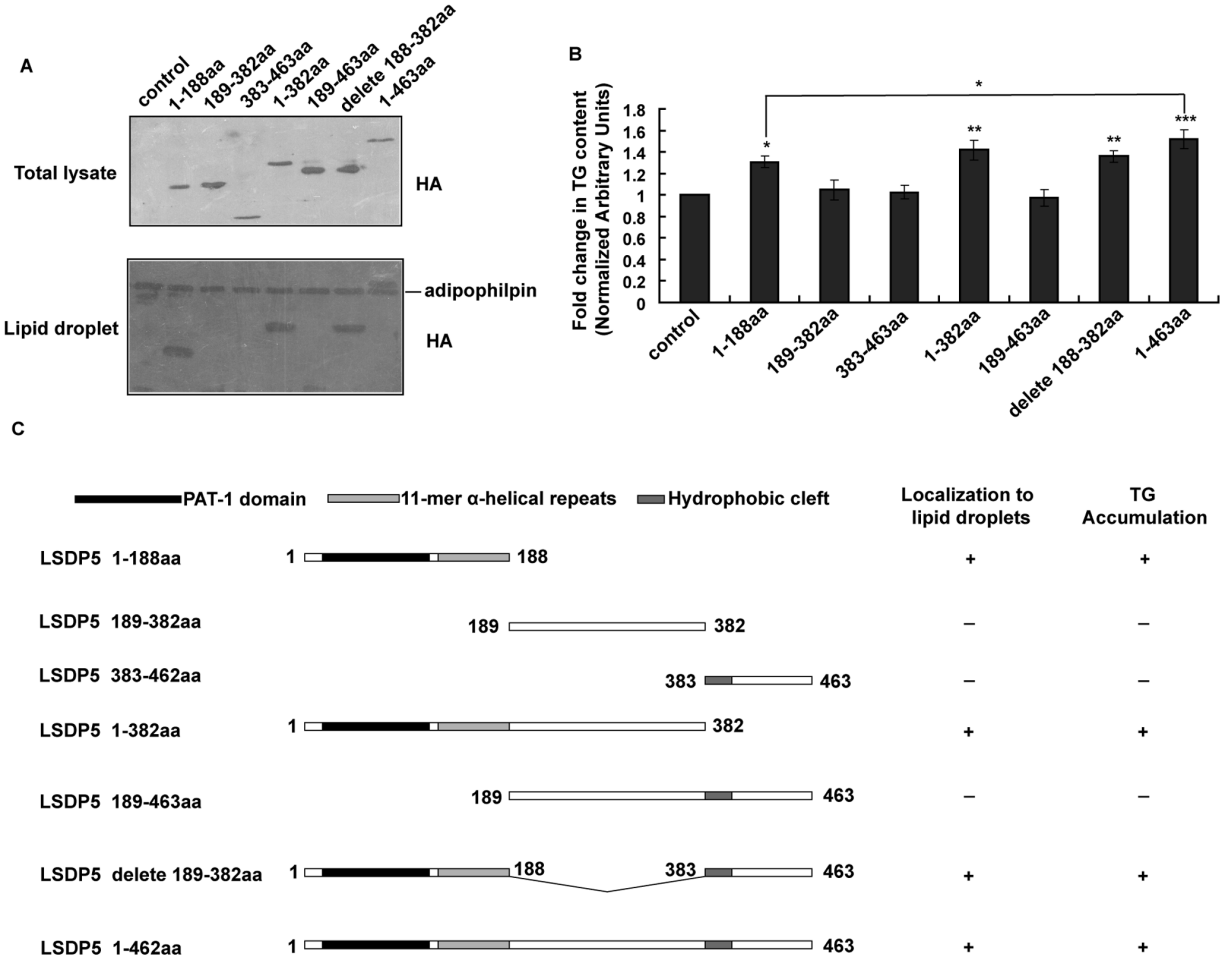
Effect of LSDP5 constructs on lipid accumulation. (A) 293T cells transfected with truncated HA-LSDP5 were incubated with 100 µM oleate overnight to enlarge the lipid droplets. Upper panel: The total lysates were subjected to Western blot analysis. An anti-HA antibody was used to show the expression levels of the HA-LSDP5 truncations used in the experiment. Lower panel: the lipid fraction was isolated by subcellular fractionation and analyzed by immunoblotting with HA and adipophilin antibodies. (B) The amount of TGs in cells transfected with truncated LSDP5 was quantified using a TG test kit. The TG content under each condition was normalized to the cellular protein level and expressed as a fold change compared with the control (pCMV5-HA). Values were normalized to 1.0. Data are presented as the mean±SEM (n = 5), ^*^ *P*<0.05, ^**^ *P*<0.01, ^***^ *P*<0.001 (Dunnett’s post hoc test following a one-way ANOVA). (C) Schematic illustration of the roles of truncated forms of LSDP5 in lipid targeting and lipid accumulation.

The function of proteins can often be ascribed to specific domains. To determine the domains of LSDP5 involved in TG accumulation and lipid droplet localization, we constructed truncations of LSDP5 according to structural analysis of hydrophobicity and sequence comparisons with other PAT members [11], [19]. An HA tag was fused to the N-terminal start codon of mouse LSDP5, and this construct was transiently transfected into 293T cells that were subsequently labeled with an anti-HA antibody. Consistent with previous reports [11], [12], the truncated proteins containing the N-terminal region of LSDP5 (1–188 aa) were observed in the lipid fraction and the proteins lacking this region appeared to lose their capacity to target lipid droplets (Figure 8A). Using a TG test kit, we determined that the TG content of cells containing LSDP5 (1–188 aa), LSDP5 (1–382 aa), LSDP5 delete (188–382 aa) (LSDP5 construct with amino acids 188 382 deleted), and LSDP5 (1–463 aa) was significantly higher than in other groups (Figure 8B) (*P*<0.05). LSDP5 constructs containing residues 1–188 showed more lipid clustering. This observation suggests that the N-terminal region of LSDP5, which contains a PAT-1 domain and 11-mer α-helical repeats, is critical for the lipid droplet localization of LSDP5 and directs TG accumulation (Figure 8C).

The TG content of cells with full-length LSDP5 (1–463 aa) was much higher than that of cells expressing LSDP5 (1–188 aa) (Figure 8B), indicating that the other domains of LSDP5 might also play roles in TG accumulation.

## Discussion

Both the formation and break-down of intracellular lipid droplets are regulated by lipid droplet-associated proteins, a group of specific proteins located on the lipid droplets that play important roles in regulating lipid droplet formation, morphology, and lipolysis [3], [10]. Many proteins have been observed to be associated with lipid droplets, including the PAT family (perilipin, adipophilin, TIP47, S3-12, and LSDP5), the cell death-inducing DFF45-like effector (CIDE) family (CIDEA, CIDEB, and CIDEC/FSP27), caveolin 1, SNARE proteins, lipid-synthesizing enzymes, lipases (hormone-sensitive lipase/HSL and ATGL), and the RAB family of GTPases [6], [8], [20]. Our previous studies have shown that two lipid droplet-associated proteins, CIDEB and CIDEC, play important roles in lipid homeostasis, whereas CIDEB mediates very low-density lipoprotein (VLDL) lipidation and maturation, and CIDEC influences the differentiation of human adipocytes [21], [22], [23]. In this study, we examined the cellular localization and physiological functions of LSDP5 in liver cells and confirmed that LSDP5 is targeted to the surface of lipid droplets and promotes TG accumulation by regulating lipolysis and fatty acid β-oxidation.

Using an immunofluorescence assay and subcellular fractionation, we demonstrated that LSDP5 is localized to lipid droplets in hepatocytes (Figure 1). The domains directing lipid droplet targeting and clustering overlaps and are localized to the 188 residues at the N-terminus of LSDP5 (Figure 8). Amino acids at the C-terminus also function in lipid accumulation (Figure 8B).

The expression of LSDP5 is sustained at an increased level under lipid loading (Figure 2), which indicates that LSDP5 might continuously affect the formation of lipid droplets and contribute to their enlargement and accumulation. To test this hypothesis, the effects of overexpressing and silencing LSDP5 were investigated in the hepatic cell line AML12 and in primary hepatocytes by loss-of-function and gain-of-function studies. Similar to the results in COS-7 and OP9 cells [13], overexpression of LSDP5 increases TG accumulation in liver cells (Figure 3). In contrast, the suppression of LSDP5 decreases TG content in liver cells (Figure 4). These data demonstrate that LSDP5 plays an important role in TG accumulation.

The specific mechanism responsible for TG accumulation mediated by LSDP5 is unclear. Our results revealed that depletion of LSDP5 results in increased TG lipolysis in hepatocytes (Figure 5&6). To clarify whether the increased TG lipolysis is due to changes in re-esterification, triacsin C was used to block the effects of long chain fatty acyl CoA synthetase (LCFACoAS) and isolate the effects of TG hydrolysis on lipolysis. The results demonstrate that silencing of LSDP5 mainly affects TG hydrolysis but has little effect on re-esterification. TG hydrolysis requires lipase binding and activation at the lipid droplet water/oil interface [3], [24]. It has been reported that LSDP5 interacts with lipase HSL, ATGL and its protein activator, α-β hydrolase domain-containing 5 (Abhd5) on lipid droplet surfaces [25], [26], [27]. The interaction of ATGL with LSDP5 decreases lipolysis [28]. Moreover, the liver has been reported to lack HSL, and ATGL is considered the most important lipases in liver cells [18]. Thus, we hypothesize that LSDP5 silencing enhances lipolysis by regulating ATGL activity. Interestingly, we observed that the mRNA level of ATGL is increased in hepatocytes when LSDP5 is silenced (Figure 5D) and that the protein level of ATGL shows a mild increase in the total lysate. In contrast, the level of ATGL protein localized to lipid droplets decreases in LSDP5-silenced cells (Figure 5E). Given that LSDP5 is no longer controlling the concentration of ATGL on the droplets, the increased level of ATGL expression might be a compensatory effect in response to the inability of LSDP5 to localize to lipid droplets. These results do not support the hypothesis that ATGL is involved in lipolysis during LSDP5 deficiency. Additional studies will be required to verify the potential players in lipolysis upon loss of LSDP5. It will also be interesting to investigate if the ATGL-LSDP5 interaction is domain-specific for LSDP5 and what roles ATGL plays when LSDP5 is depleted.

In addition, we determined that fatty acid β-oxidation in the mitochondria is up-regulated when LSDP5 is knocked down (Figure 6). It remains unclear whether the increase in the level of fatty acid oxidation is a direct effect of LSDP5 deficiency or an indirect result. Using the PPARα inhibitor GW6471, we showed that PPARα is required for the increase in the level of fatty acid oxidation in LSDP5-deficient cells, implying that LSDP5 indirectly affects fatty acid oxidation.

TG synthesis is also a critical metabolic pathway contributing to the lipid content in cells. The rate of TG synthesis is not changed when LSDP5 is down-regulated (Figure 5A). In vivo, the de novo synthesis of fatty acids is primarily regulated by ACC1 and FAS. TG synthesis is regulated by different enzymes, such as ACS and AGPAT. However, we did not detect significant changes in the transcription levels of these enzymes (Figure 5D). It is unlikely that LSDP5 has a direct effect on TG synthesis because the expression of LSDP5 can be induced in liver cells either by fasting (fat mobilization) [13] or administration of free fatty acids. The effect of LSDP5 on the secretion of TG from the liver is an area of active investigation.

It seems paradoxical that PPARα (which stimulates lipolysis and fatty acid oxidation) induces LSDP5 (which functions to limit lipolysis). Most PAT genes are transcriptionally regulated by PPARs; S3-12 and perilipin are regulated by PPARγ; adipophilin is regulated by PPARα and PPARβ/δ; and TIP47 does not appear to be regulated by PPARs. In the liver, the transcription of LSDP5 is regulated by PPARα [15]. However, all PAT proteins, with the exception of S3-12, have been observed to prevent the lipolysis of lipid droplets [6], [11], [12], [13]. These observations allow us to speculate that PAT family proteins determine the properties of lipid droplets in terms of the storage and mobilization of lipids. This function has been most extensively studied for perilipin, which inhibits lipolysis in its non-phosphorylated form and stimulates lipolysis when phosphorylated [6], [10]. Based on the current functional data [6], [16], and the high degree of primary sequence similarity among PAT family members [12], [13], [14], [19], it is likely that LSDP5 could also serve as a regulator of both consumption and accumulation of lipids in the liver, which would be similar to perilipin in adipose tissue. A more comprehensive study on the dual role of LSDP5 involving amino acid sequence analysis and protein-protein interactions is currently being performed to address this hypothesis. Our data also demonstrate that PPARα is activated when the expression of LSDP5 is silenced, which suggests that LSDP5 might not only be a downstream target of PPARα trans-activation, but may also be involved in a feedback-sensing pathway. Therefore, the levels of PPARα and LSDP5 may have a reciprocal influence on each other and be maintained in a dynamic balance.

### Conclusion

Our findings suggest that LSDP5 is a novel regulator in controlling lipid homeostasis in hepatocytes. It may play an important role in lipid accumulation by regulating lipolysis and influencing fatty acid β-oxidation through PPARα activation. LSDP5 could serve as a potentially important therapeutic target for the treatment of non-alcoholic fatty liver disease. However, further studies are necessary to elucidate the role of LSDP5 in hepatic steatosis in vivo.

## Materials and Methods

### Ethics Statement

The animal experiments in this study were performed in accordance with the Guide for the Care and Use of Laboratory Animals of the National Institutes of Health and approved by the Ethical Committee of Fourth Military Medical University (Permit number:SCXK2007-007). All surgery was performed under sodium pentobarbital anesthesia, and all efforts were made to minimize animal suffering.

### Antibodies and Reagents

The affinity-purified rabbit polyclonal LSDP5 antibody was generated as previously described [29], [30] and was also purchased from Thermo Scientific (catalog no.PT-46215, Bonn, Germany). The specificities of the LSDP5 antibodies were confirmed by Western blot analysis (Figure S4). Guinea pig polyclonal anti-adipophilin (catalog no. RDI-PROGP40) was obtained from Research Diagnostics Inc (New Jersey, USA). Monoclonal HA antibody (catalog no. H9658) and monoclonal α-tubulin antibody (catalog no. T9026) were purchased from Sigma (St. Louis, USA). Rabbit polyclonal PPARα antibody (catalog no. sc-9000), goat polyclonal CPT1α antibody (catalog no. sc-20514) and rabbit polyclonal ACO antibody (catalog no. sc-98499) were obtained from Santa Cruz Biotechnology (California, USA). Mouse monoclonal His antibody (catalog no. 34698) was purchased from Qiagen (Valencia, USA). Rabbit polyclonal ATGL antibody (catalog no. #2138) was obtained from Cell Signaling (Beverly, USA). Cy3-conjugated anti-mouse IgG (catalog no. A10521) was purchased from Invitrogen (Carlsbad, USA). Collagenase type II was obtained from Sigma. Fatty acid-free bovine serum albumin (BSA) was purchased from Calbiochem (La Jolla, USA) and oleate was obtained from Sigma. BODIPY 493/50, Nile red and MitoTracker Red CMXRos were purchased from Invitrogen (Carlsbad, USA). [9, 10^−3^H] oleate and [2^−3^H] glycerol was obtained from Amersham Pharmacia Biotech (Milan, Italy), and scintillation liquid (OptiFluor) was purchased from Perkin Elmer (Boston, USA). Thin-layer chromatograph (TLC) plates were obtained from Merck (Darmstadt, Germany), and the internal standards cholesterol, cholesteryl oleate, phosphatidylcholine and TG were purchased from Sigma. Selective PPARα agonist WY 14643 and PPARα antagonist GW6471 were obtained from Sigma.

### Cell Culture

AML12 cells (American Type Culture Collection, Manassas, USA) were cultured in DMEM/Ham’s F12 medium supplemented with 10% fetal bovine serum (FBS) (5 µg/ml insulin, 5 µg/ml transferrin, 5 µg/ml selenium, 40 ng/ml dexamethasone, 100 units/ml penicillin and 100 µg/ml streptomycin). Cell culture reagents and FBS were purchased from GIBCO (Gland Island, USA).

Oleate-containing media was prepared as previously described [31]. Briefly, oleate was dissolved in ethanol to a concentration of 200 mM and then combined with 10% fatty acid-free BSA (5 mM). The pH of the solution was adjusted to 7.5, and the oleate stock solution was filter-sterilized and stored at −20°C. A control solution containing ethanol and BSA was similarly prepared. For individual experiments, the culture medium was removed and replaced by 2% FBS-medium containing appropriate stock solutions.

### Mouse Hepatocyte Isolation

Mouse hepatocytes were isolated using a two-step in situ collagenase perfusion procedure as previously described [22]. Six-week-old C57BL/6 mice were purchased from the Fourth Military Medical University Animal Center. The livers from the C57BL/6 mice were perfused in situ through the portal vein with EGTA buffer (0.5 mM EGTA, 137 mM NaCl, 4.7 mM KCl, 1.2 mM KH_2_PO_4_, 0.65 mM MgSO_4_, and 10.07 mM HEPES at pH 7.4) at a flow rate of 5 ml/min for 10 min, followed by collagenase buffer (67 mM NaCl, 6.7 mM KCl, 4.76 mM CaCl_2_, 0.035% collagenase type II, and 10.07 mM HEPES at pH 7.6) at a flow rate of 5 ml/min for 15 min. After centrifugation, the hepatocytes were collected and seeded in DMEM containing 10% FBS, 100 units/ml penicillin, and 100 µg/ml streptomycin.

### Construction of Expression Plasmids

The full-length and truncated forms of LSDP5 were PCR amplified using specific primers (Table S1) corresponding to the regions described in Figure 8C. All of the PCR products contained a NedI or NcoI restriction site at the 5′-end and a BglII restriction site at the 3′-end. The PCR products were digested with NedI/NcoI and BglII and inserted in-frame into pBluescript KS-HA to generate HA-tagged constructs. The HA-tagged inserts were digested with HindIII and XbaI and subcloned into the pCMV5 vector to produce the mammalian expression constructs. The sequences of all of the constructs were confirmed by DNA sequencing.

### Depletion of LSDP5 in AML12 Cells

The siRNA constructs used to target LSDP5 mRNA were designed using siRNA TARGET FINDER software (http://www4.appliedbiosystems.com/techlib/misc/siRNA_finder.html). The sense oligonucleotide was 5′-GGCAAGCACACAATGATGC-3′. The specificity of the LSDP5-siRNA is shown in Figure S5. Oligonucleotides encoding the siRNAs were inserted into the pSilencer 3.1-H1 neo vector (kindly provided by Dr. Peng Li, Tsinghua University, China), and the resulting construct was transfected into AML12 cells using Lipofectamine 2000 (Invitrogen, Carlsbad, USA). Approximately 48 h after transfection, selection medium containing G418 (500 µg/ml) was used to culture cells for 20 d. The isolated G418-resistant cell clones were then selected and amplified. An siRNA sequence specific for the GL2 luciferase gene was used as the control siRNA [32].

### Generation of Recombinant Adenovirus

The recombinant adenovirus carrying full-length LSDP5 with an HA epitope tag on the N-terminal end was constructed using the AdEasy-1 System (Stratagene, La Jolla, USA). The adenovirus carrying green fluorescent protein (GFP) (Benyuan Zhengyang Gene Technology Company Ltd., Beijing, China) was used as a control. Adenovirus-mediated siRNA of LSDP5 was generated using the same method. After large-scale amplification of AD293 cells, recombinant adenovirus was purified using CsCl density-gradient ultracentrifugation, dialyzed against PBS supplemented with 15% glycerol, and stored at -80°C. To infect cells, Ad-LSDP5 and Ad-GFP (control virus) or Ad-si-LSDP5 and Ad-si-GL2 luciferase (si-control virus) were directly added to primary cultured hepatocytes or AML12 cells.

### Lipid Staining and Mitochondria Staining

Cells were grown on coverslips in 12-well plates. Cell lipids were stained with Nile red or BODIPY 493/503 as previously described [22], [23]. MitoTracker Red CMXRos was used to stain the mitochondria according to the manufacturer’s instructions.

### Isolation of Lipid Droplets by Subcellular Fractionation

Lipid droplet fractions were isolated by sucrose gradients as previously described [22]. Briefly, cells (three 150-mm dishes) were washed with PBS, collected by centrifugation, resuspended in a hypotonic medium (10 mM HEPES/NaOH (pH 7.4), 1 mM EDTA, 10 mM sodium fluoride, and protease inhibitor mixture and incubated for 10 min on ice followed by 10 strokes with a Dounce homogenizer. The lysate was mixed with an equal volume of disruption buffer containing 1.08 M sucrose. The homogenates were centrifuged to remove the nuclei, and the supernatant was overlaid with 2 ml each of 0.27 M sucrose buffer, 0.13 M sucrose buffer, and top buffer (25 mM Tris HCl, 1 mM EDTA, and 1 mM EGTA). The gradient was centrifuged at 250,000 g 1 h at 4°C in a Beckman XP-100 ultracentrifuge. After centrifugation, the buoyant lipid droplet fractions were collected on the top of the gradient. The floating lipid droplet fractions were harvested by careful suctioning with Pasteur pipets. The proteins on lipid droplets were precipitated by ice-cold acetone and washed twice with acetone/diethylether (1∶1, vol/vol).

### Lipid Analysis

To measure the total TG level, lipids were extracted from cells using the Folch method [33]. Dried lipids were reconstituted in chloroform:methanol (2∶1, v/v) and assayed using a TG test kit (WAKO Chemicals, Osaka, Japan). The TG content was normalized to the protein content and measured using the Bio-Rad Protein assay (Bio-Rad, Hercules, USA).

AML12 cells were infected with an adenovirus carrying LSDP5 siRNA for 24 h and incubated in experimental medium containing 1 µCi/ml [2^−3^H] glycerol or 1 µCi/ml [9, 10^−3^H] oleate (Amersham Pharmacia Biotech, Milan, Italy). The amounts of tritium incorporated into the TGs was monitored at 2 h, 4 h, 8 h, and 12 h. Lipids were extracted from cells using chloroform:methanol (1∶2; v/v) and were separated using TLC [23]. The TG spots were scratched off the TLC plates, dissolved in 500 µl of methanol:water (1∶2; v/v) and counted in 5 ml of scintillation liquid (OptiFluor) using a beta counter (LS 8000, Beckman Instruments). To measure cellular lipolysis, cells were incubated in 24-well plates and were treated overnight with 0.4 µCi/well [9, 10^−3^H] oleate. Following the 24 h loading period, cells were washed three times with sterile PBS (pH 7.4) and placed in an efflux medium consisting of DMEM/Ham’s F12 medium that included 1% fatty acid-free BSA as a fatty acid acceptor. The efflux of radioactivity into the medium was measured over time. Re-esterification of the fatty acids was prevented by inclusion of 2.5 µM triacsin C (Santa Cruz, California, USA), an inhibitor of acetyl co-enzyme A synthetase. Mitochondrial β-oxidation of [9,10^−3^H] oleate in AML12 cells was assayed by the degree of incorporation of ^3^H into H_2_O using a liquid scintillation counter [34]. The results are expressed as disintegrations per minute (DPM) and are normalized to the protein concentration.

### Copy Number of mtDNA

Total DNA was extracted using the Universal Genomic DNA Extraction Kit (TaKaRa, Tokyo, Japan). The copy number of mtDNA was determined using real-time PCR as previously reported [35]. The primers used to assay NADH dehydrogenase subunit I were sense, 5′- CCCATTCGCGTTATTCTT-3′ (sense), and antisense, 5′- AAGTTGATCGTAACGGAAGC-3′ (antisense). The lipoprotein lipase gene was used as a reference for nuclear DNA quantification and the primers used were sense, 5′- GGATGGACGGTAAGAGTGATTC-3′(sense), and antisense, 5′- ATCCAAGGGTAGCAGACAGGT -3′(antisense).

### RNA Extraction and Quantitative Real-Time PCR

Procedures for RNA extraction and real-time PCR analysis have been previously described [7]. In short, the total RNAs of tissues or cells was extracted using Trizol reagent according to the manufacturer’s directions (Invitrogen, CA, USA). Reverse-transcript PCR was performed with the reverse transcription kit (TaKaRa, Shiga, Japan). Primer sequences for real-time PCR analysis are listed in Table S2. Real-time PCR reaction components were derived from the SYBR Green Kit (TaKaRa, Shiga, Japan). PCR products were quantified ﬂuorometrically using SYBR Green, and normalized to the housekeeping gene GAPDH and relative to the control group according to the following formula: target amount = 2^−△△Ct^, where −△△Ct = {[Ct (target gene)-Ct (GAPDH)]-[Ct (control)-Ct (GAPDH control)}.

### Immunofluorescence Assay and Western Blotting

Immunofluorescence analyses were performed on cells grown on coverslips. The cells were fixed in a freshly prepared solution of 4% paraformaldehyde, rinsed, and permeabilized with 0.1% Triton X-100. Permeabilized cells were incubated with 5% goat serum in PBS to block non-specific binding. After thorough rinsing with PBS, the cells were incubated 24–48 h at 4°C with a rabbit anti-HA antibody (diluted 1∶5000, Sigma) and incubated with a Cy3-conjugated anti-mouse antibody (diluted 1∶500, Invitrogen). Specimens were observed using a Zeiss 200 M fluorescence microscope, and images were captured with an AxioCam MRm camera and Axio Vision software 4.5 (Zeiss). The images of co-localization images were captured using laser-scanning confocal microscopy (FV-300/IX71, Olympus, Tokyo, Japan).

Western blots were performed as previously described [36]. The working dilutions of primary antibodies were as follows: HA-antibody (Sigma), 1∶5000; LSDP5 antibody (made by our lab), 1∶1000; LSDP5-antibody (Thermo), 1∶400; PPARα (Santa Cruz), 1∶500; adipophilin (RDI), 1∶2000; CPT1α (Santa Cruz), 1∶400; ACO (Santa Cruz), 1∶200; α-tubulin (Sigma), 1∶10,000; and His (Qiagen), 1∶5000.

### PPARα Activity Assay

Nuclear extracts were prepared according to the kit instructions and stored at -80°C until analysis. Activated PPARα from the nuclear extract was measured by its DNA binding to an immobilized oligonucleotide containing a PPAR consensus binding site using the TransBinding PPARα Assay Kit (Panomics, Redwood City, USA). Binding was assessed by measuring the absorbance at 450 nm.

### Statistical Analysis

Results are expressed as the mean±SEM from the indicated number of experiments. Data were analyzed with the paired-sample two-sided Student’s *t*-tests for paired samples and one-way ANOVA tests and Dunnett’s post hoc tests for comparisons of multiple groups. All statistical analyses were performed using SPSS version 11.0 (SPSS Inc., Chicago, USA). A probability level of 0.05 was considered significant.

## Supporting Information

Figure S1 **LSDP5 overexpression enhanced the cellular lipid content in primary mouse hepatocytes.** Primary mouse hepatocytes were infected with an adenovirus containing HA-tagged LSDP5 for 6 h and then incubated with 200 µM oleate for 24 h. (A) LSDP5 overexpression in mouse primary hepatocytes was verified by Western blot. (B) Up-regulation of LSDP5 mRNA in primary mouse hepatocytes. Data are presented as the mean±SEM (n = 4), ^*^ *P*<0.05. (C) Representative photos showing LSDP5 overexpression in primary mouse hepatocytes. Neutral lipids were stained with BODIPY 493/503 and nuclei were labeled with Hoechst 33258. Scale bar = 5 µm. (D) The TG content in primary mouse hepatocytes overexpressing LSDP5. Data are presented as the mean±SEM (n = 5), ^*^ *P*<0.05. Data in this figure were analyzed with paired Student’s *t* tests.
(TIF)

Figure S2 **Suppression of LSDP5 affects lipid storage in primary mouse hepatocytes.** Primary mouse hepatocytes were infected with adenovirus containing siRNA against LSDP5 for 24 h and incubated with 200 µM oleate overnight. (A) After infection and lipid loading, Western blotting revealed that the adenovirus-mediated silencing of LSDP5 effectively reduced the LSDP5 protein level (at least 90%) from primary mouse hepatocytes. Similar results were obtained from three independent experiments. (B) Knock-down of LSDP5 mRNA in primary mouse hepatocytes was assessed with real-time PCR. Data are presented as the mean±SEM (n = 4), ^*^ *P*<0.05. (C) Representative photos showing LSDP5 depletion in primary mouse hepatocytes. Neutral lipids were stained with BODIPY 493/503, and nuclei were labeled with Hoechst 33258. Scale bar = 5 µm. (D) The TG content of primary mouse hepatocytes expressing a siRNA targeting LSDP5. Data are presented as the mean±SEM (n = 5), ^*^ *P*<0.05. Data in this figure were analyzed with paired Student’s *t* tests.
(TIF)

Figure S3 **Lipid storage was blocked in AML12-si-LSDP5 cells.** (A) The plasmid pSilencer3.1-H1 neo containing siRNA against LSDP5 was transfected into AML12 cells and was followed by G418 selection. Two stable clones were selected and are referred to as AML12-si-LSDP5 (22) and AML12-si-LSDP5 (37). Western blot analysis was performed on AML12 cells, AML12-si-control cells, AML12-si-LSDP5 (22) cells and AML12-si-LSDP5 (37) cells using an LSDP5 antibody. Immunoblot analysis revealed that the expression of LSDP5 was significantly reduced in the two selected stable clones, especially in AML12-si-LSDP5 (37) cells. The expression levels of LSDP5 are expressed as a ratio to α-tubulin (representative of four experiments). Data are presented as the mean±SEM, ^*^ *P*<0.05 (Dunnett’s post hoc test following a one-way ANOVA). AML12-si-LSDP5 (37) cells were used in the following experiments and are referred to as AML12-si-LSDP5 for short. (B) Verification of the LSDP5 silencing using real-time PCR. Data are presented as the mean±SEM (n = 4), ^*^ *P*<0.05. (C) AML12-si-control cells and AML12-si-LSDP5 cells were incubated with 200 µM oleate for 24 h and stained with BODIPY 493/503. Scale bar = 15 µm. (D) The TG content of AML12-si-control cells and AML12-si-LSDP5 cells after oleate supplementation. Data are presented as the mean±SEM (n = 5),^ *^ *P*<0.05 (paired Student’s *t* test).
(TIF)

Figure S4 **The specificity of the LSDP5 antibody.** 293T cells were transfected with pCMV5-HA-LSDP5 encoding full-length (1–463 aa) LSDP5 (HA-LSDP5) or pCMV5-HA-LSDP5 encoding the carboxy-terminal domain (189–463 aa) of LSDP5 (HA-LSDP5-Ct). Western blotting was performed using an anti-HA antibody, a commercial LSDP5 antibody (LSDP5 Ab1) and an LSDP5 antibody generated in this study (LSDP5 Ab2).
(TIF)

Figure S5 **The specificity of LSDP5-siRNA.** 293T cells were transfected with pCMV5-HA-LSDP5 (A), pCMV5-HA-perilipin (B), pCMV5-HA-adipophilin (C), psilencer-si-LSDP5 and psilencer-si-control as indicated. The expression level of LSDP5, perilipin or adipophilin was analyzed by Western blotting with an anti-HA antibody. Each experiment was repeated at least 3 times.
(TIF)

Table S1 **Primer sequences for the full-length and truncated forms of LSDP5.**
(DOC)

Table S2 **Primer sequences for real-time PCR.**
(DOC)
