# Supplementary figures and images for: LSDP5 Enhances Triglyceride Storage in Hepatocytes by Influencing Lipolysis and Fatty Acid β-Oxidation of Lipid Droplets
Source: PLoS One. 2012 Jun 1;7(6):e36712. doi: 10.1371/journal.pone.0036712 (PMC3365886; doi:10.1371/journal.pone.0036712)

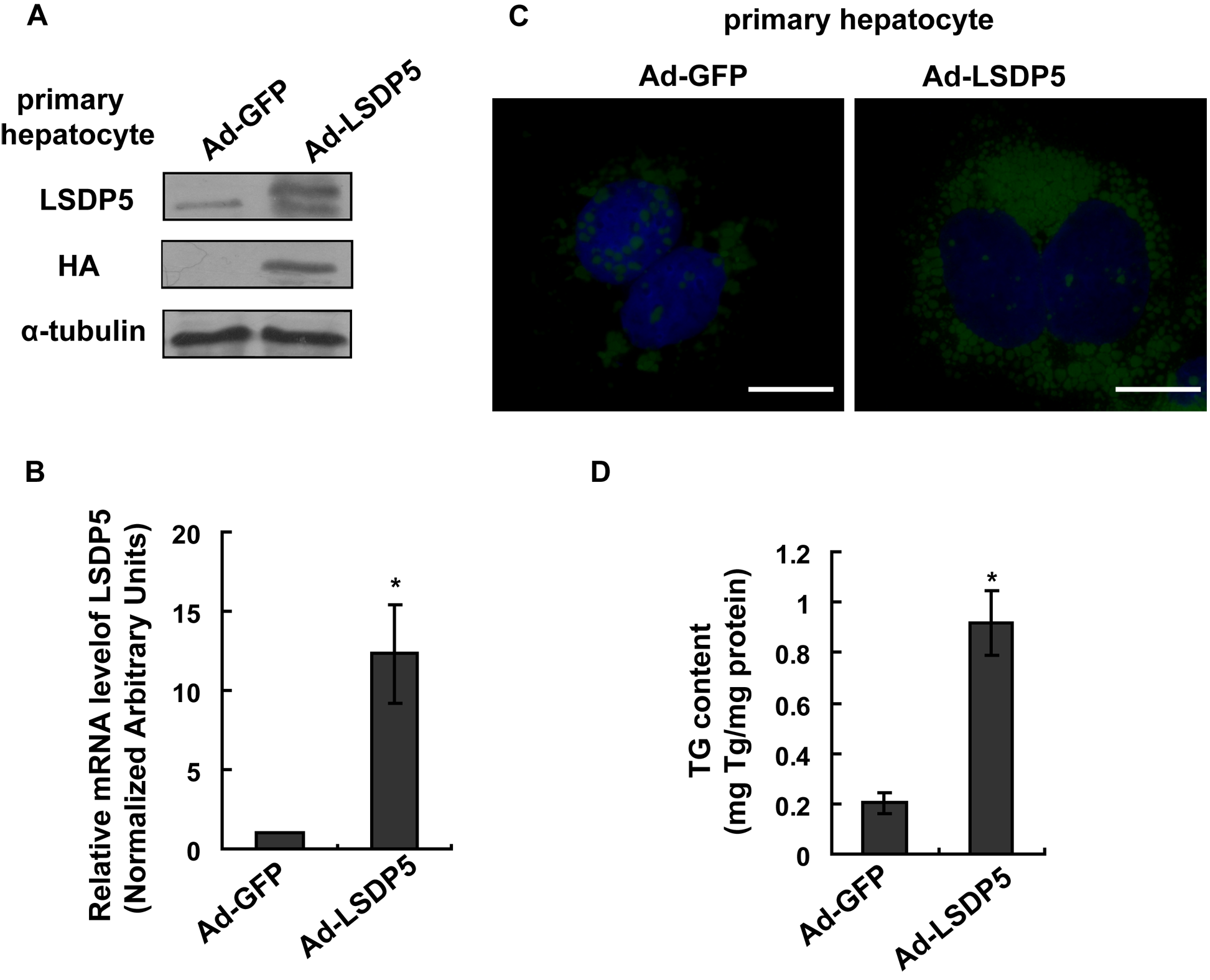

Supplement: Figure S1 — LSDP5 overexpression enhanced the cellular lipid content in primary mouse hepatocytes. Primary mouse hepatocytes were infected with an adenovirus containing HA-tagged LSDP5 for 6 h and then incubated with 200 µM oleate for 24 h. (A) LSDP5 overexpression in mouse primary hepatocytes was verified by Western blot. (B) Up-regulation of LSDP5 mRNA in primary mouse hepatocytes. Data are presented as the mean±SEM (n = 4), * P<0.05. (C) Representative photos showing LSDP5 overexpression in primary mouse hepatocytes. Neutral lipids were stained with BODIPY 493/503 and nuclei were labeled with Hoechst 33258. Scale bar = 5 µm. (D) The TG content in primary mouse hepatocytes overexpressing LSDP5. Data are presented as the mean±SEM (n = 5), * P<0.05. Data in this figure were analyzed with paired Student’s t tests. (TIF) [file pone.0036712.s001.tif]

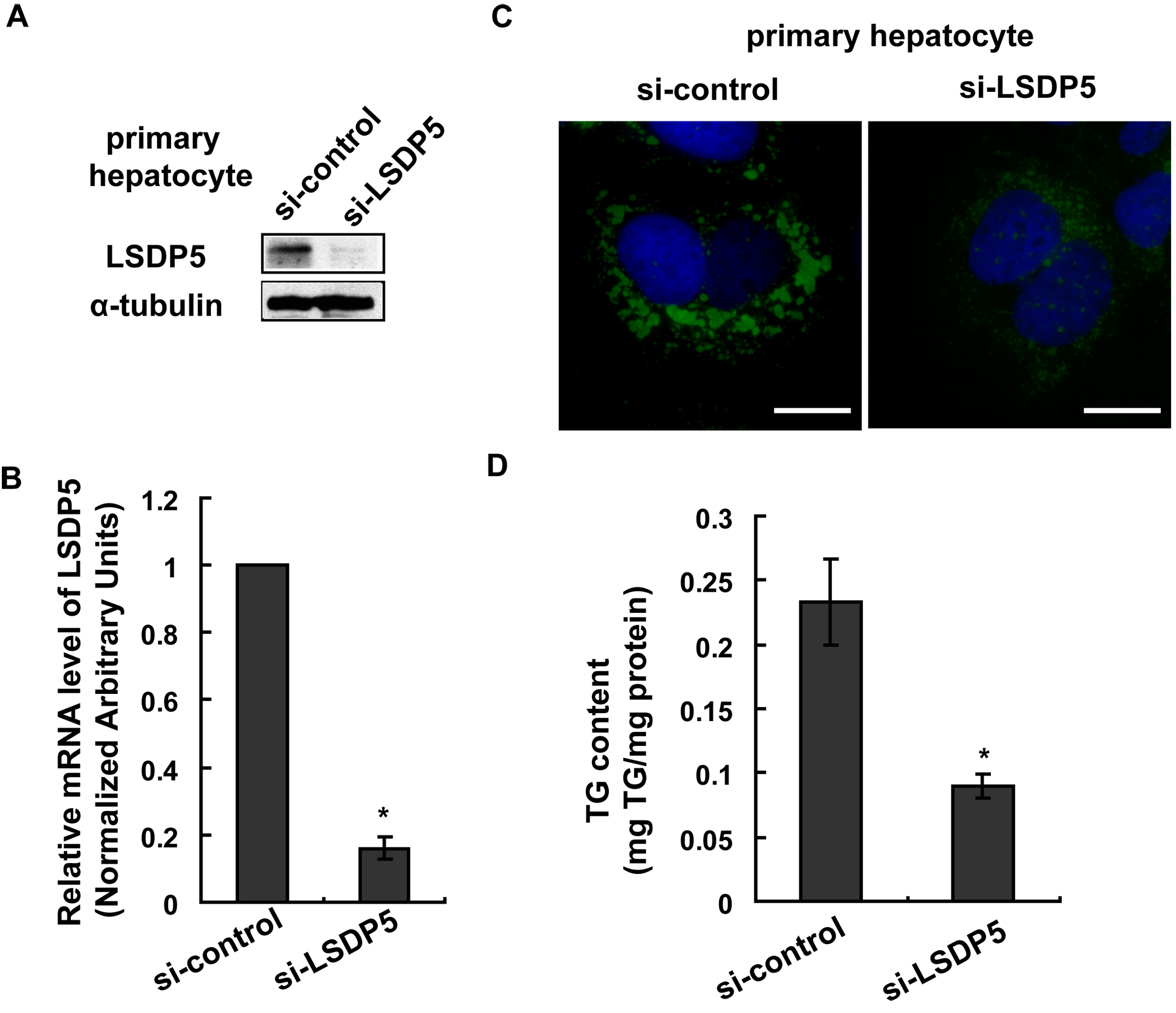

Supplement: Figure S2 — Suppression of LSDP5 affects lipid storage in primary mouse hepatocytes. Primary mouse hepatocytes were infected with adenovirus containing siRNA against LSDP5 for 24 h and incubated with 200 µM oleate overnight. (A) After infection and lipid loading, Western blotting revealed that the adenovirus-mediated silencing of LSDP5 effectively reduced the LSDP5 protein level (at least 90%) from primary mouse hepatocytes. Similar results were obtained from three independent experiments. (B) Knock-down of LSDP5 mRNA in primary mouse hepatocytes was assessed with real-time PCR. Data are presented as the mean±SEM (n = 4), * P<0.05. (C) Representative photos showing LSDP5 depletion in primary mouse hepatocytes. Neutral lipids were stained with BODIPY 493/503, and nuclei were labeled with Hoechst 33258. Scale bar = 5 µm. (D) The TG content of primary mouse hepatocytes expressing a siRNA targeting LSDP5. Data are presented as the mean±SEM (n = 5), * P<0.05. Data in this figure were analyzed with paired Student’s t tests. (TIF) [file pone.0036712.s002.tif]

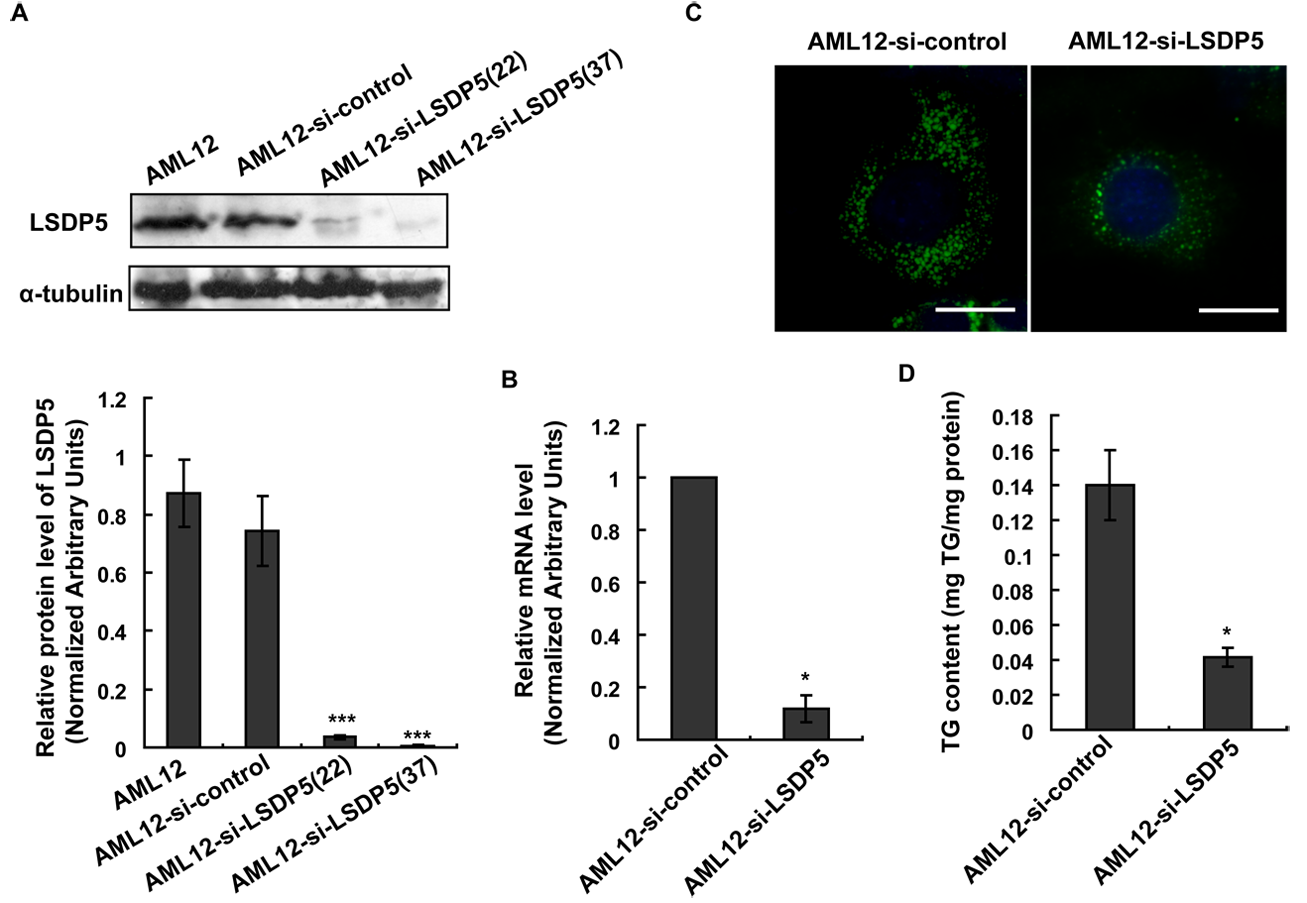

Supplement: Figure S3 — Lipid storage was blocked in AML12-si-LSDP5 cells. (A) The plasmid pSilencer3.1-H1 neo containing siRNA against LSDP5 was transfected into AML12 cells and was followed by G418 selection. Two stable clones were selected and are referred to as AML12-si-LSDP5 (22) and AML12-si-LSDP5 (37). Western blot analysis was performed on AML12 cells, AML12-si-control cells, AML12-si-LSDP5 (22) cells and AML12-si-LSDP5 (37) cells using an LSDP5 antibody. Immunoblot analysis revealed that the expression of LSDP5 was significantly reduced in the two selected stable clones, especially in AML12-si-LSDP5 (37) cells. The expression levels of LSDP5 are expressed as a ratio to α-tubulin (representative of four experiments). Data are presented as the mean±SEM, * P<0.05 (Dunnett’s post hoc test following a one-way ANOVA). AML12-si-LSDP5 (37) cells were used in the following experiments and are referred to as AML12-si-LSDP5 for short. (B) Verification of the LSDP5 silencing using real-time PCR. Data are presented as the mean±SEM (n = 4), * P<0.05. (C) AML12-si-control cells and AML12-si-LSDP5 cells were incubated with 200 µM oleate for 24 h and stained with BODIPY 493/503. Scale bar = 15 µm. (D) The TG content of AML12-si-control cells and AML12-si-LSDP5 cells after oleate supplementation. Data are presented as the mean±SEM (n = 5), * P<0.05 (paired Student’s t test). (TIF) [file pone.0036712.s003.tif]

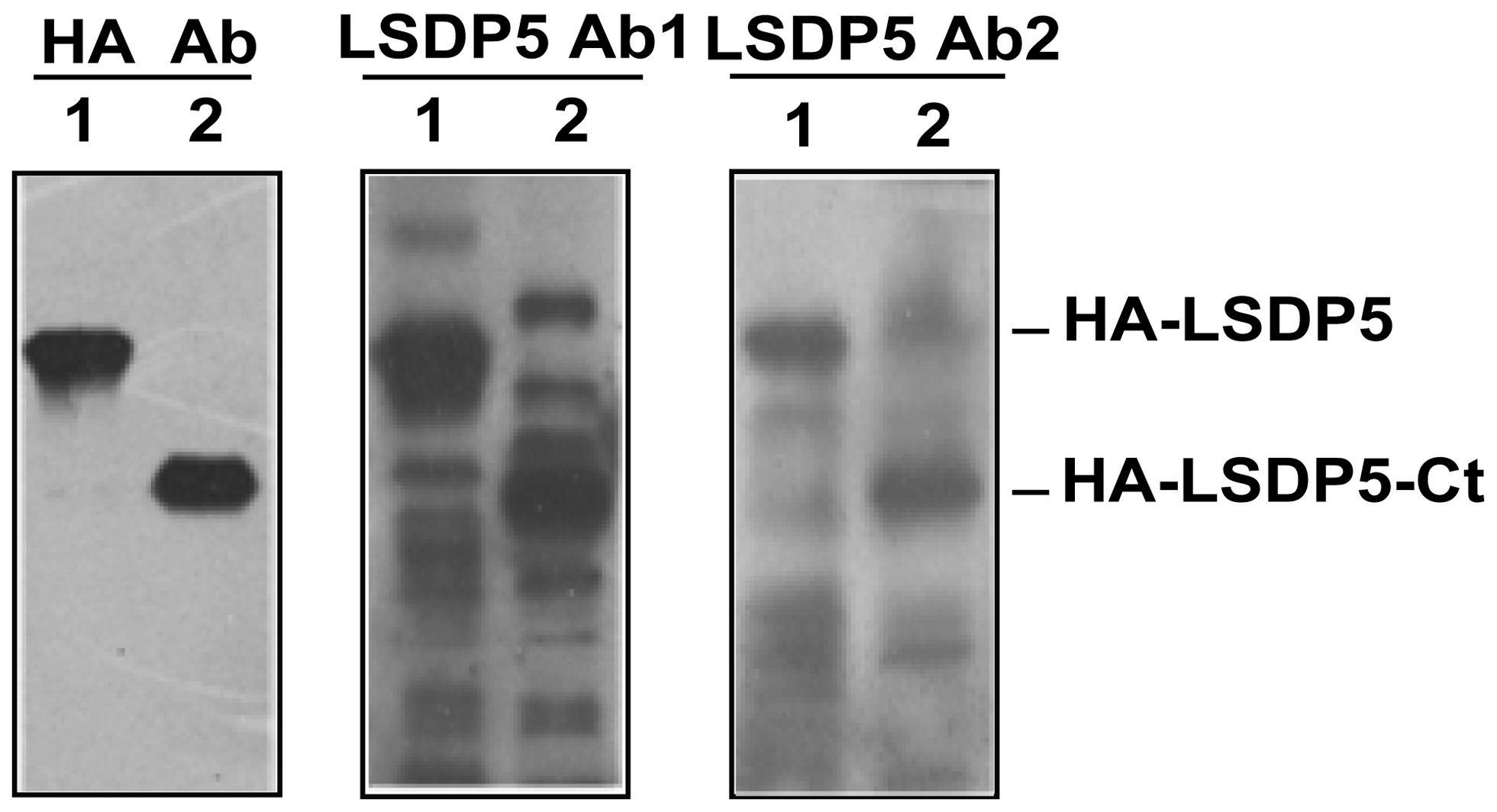

Supplement: Figure S4 — The specificity of the LSDP5 antibody. 293T cells were transfected with pCMV5-HA-LSDP5 encoding full-length (1–463 aa) LSDP5 (HA-LSDP5) or pCMV5-HA-LSDP5 encoding the carboxy-terminal domain (189–463 aa) of LSDP5 (HA-LSDP5-Ct). Western blotting was performed using an anti-HA antibody, a commercial LSDP5 antibody (LSDP5 Ab1) and an LSDP5 antibody generated in this study (LSDP5 Ab2). (TIF) [file pone.0036712.s004.tif]

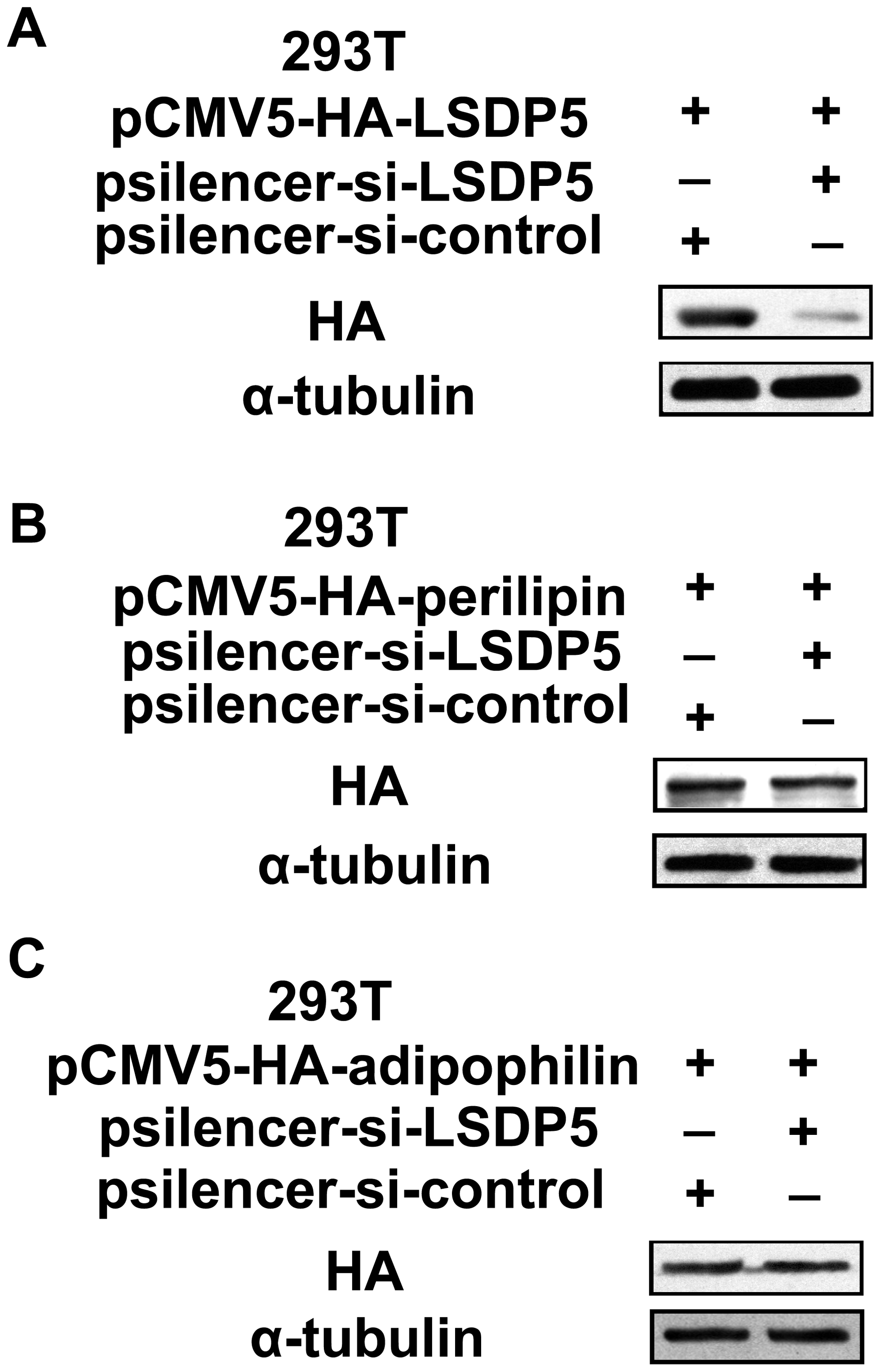

Supplement: Figure S5 — The specificity of LSDP5-siRNA. 293T cells were transfected with pCMV5-HA-LSDP5 (A), pCMV5-HA-perilipin (B), pCMV5-HA-adipophilin (C), psilencer-si-LSDP5 and psilencer-si-control as indicated. The expression level of LSDP5, perilipin or adipophilin was analyzed by Western blotting with an anti-HA antibody. Each experiment was repeated at least 3 times. (TIF) [file pone.0036712.s005.tif]
