# Supplementary material for: LSDP5 Enhances Triglyceride Storage in Hepatocytes by Influencing Lipolysis and Fatty Acid β-Oxidation of Lipid Droplets
Source: PLoS One. 2012 Jun 1;7(6):e36712. doi: 10.1371/journal.pone.0036712 (PMC3365886; doi:10.1371/journal.pone.0036712)
Supplement: Table S1 — Primer sequences for the full-length and truncated forms of LSDP5. (DOC) [file pone.0036712.s006.doc]

**Table S1.** Primer Sequences for the full-length and truncated forms of LSDP5

| **Gene Name** | **Direction** | **Oligonucleotide Sequences** |
| --- | --- | --- |
| LSDP5 | Forward | CCATATGGACCAGAGAGGTGAAGACACCAC |
| LSDP5(1-188aa) | Reverse | GAAGATCTTTATAGCTCAGCCTCAGTCATGGG |
| LSDP5(189-382aa) | Forward | CATGCCATGGCAGTCCTGGCAGCTGAGGC |
| LSDP5(189-382aa) | Reverse | GAAGATCTTACCGCAGGACCAAATCCAGGA |
| LSDP5(383-462aa) | Forward | CCATATGGCCATGCCACTGCCCTGGCTT |
| LSDP5 | Reverse | GGAAGATCTTCAGGAGTCCAGCTCTGGCA |
