# Supplementary material for: LSDP5 Enhances Triglyceride Storage in Hepatocytes by Influencing Lipolysis and Fatty Acid β-Oxidation of Lipid Droplets
Source: PLoS One. 2012 Jun 1;7(6):e36712. doi: 10.1371/journal.pone.0036712 (PMC3365886; doi:10.1371/journal.pone.0036712)
Supplement: Table S2 — Primer sequences for real-time PCR. (DOC) [file pone.0036712.s007.doc]

**Table S2. Primer Sequences for real-time PCR**

| **Gene Name** | **Direction** | **Oligonucleotide Sequences** |
| --- | --- | --- |
| Adipophlin | Forward | CTACGACGACACCCAT |
|  | Reverse | CATTGCGGAATACGGAG |
| Sdha | Forward | GTTGGCGCAGTTTCGAGGCT |
|  | Reverse | GCCGCAGGTCTGTTTTTGGA |
| ACC1 | Forward | GAGCTAGAATTGGACTTGCAG |
|  | Reverse | ACTCCCTCAAAGTCATCACAA |
| FAS | Forward | GACAGCTTCCGTGAGTCTATC |
|  | Reverse | CCTTCTTGAGAGCCTGCAGCA |
| PPARα | Forward | TCACACAATGCAATCCGTTT |
|  | Reverse | GGCCTTGACCTTGTTCATGT |
| CPT1α | Forward | TGTCCAAGTATCTGGCAGTCG |
|  | Reverse | CATAGCCGTCATCAGCAACC |
| LSDP5 | Forward | GTGATCAGACAGCTCAGGACCCT |
|  | Reverse | CGATTCACCACATTCTGCTGG |
| Cox4 | Forward | TACTTCGGTGTGCCTTCGA |
|  | Reverse | TGACATGGGCCACATCAG |
| Cox7a1 | Forward | TGGGCTGGGCCTCCTT |
|  | Reverse | GTTTGTCCAAGTCCTCCAAGCT |
| GAPDH | Forward | ACCCCTTCATTGACCTCAACTACATGG |
|  | Reverse | ATTTGATGTTAGTGGGGTCTCGCTCCT |
| ATGL | Forward | ATATCCCACTTTAGCTCCAAGG |
|  | Reverse | CAAGTTGTCTGAAATGCCGC |
| ACS | Forward | TGAAGCCATCACGATCATAGTCAAC |
|  | Reverse | TCGACTGTACTTTGTGGAAGATCA |
| AGPAT2 | Forward | TCAAGTACGTGTATGGCCTTCGCT |
|  | Reverse | TGCACACAGCGCTTAGGGAGTATT |
| ACO | Forward | ATATTTACGTCACGTTTACCCCGG |
|  | Reverse | GGCAGGTCATTCAAGTACGACAC |
